# Profile of STING agonist and inhibitor research: a bibliometric analysis

**DOI:** 10.3389/fphar.2025.1528459

**Published:** 2025-02-11

**Authors:** Xuemei Wang, Qian Wang, Yidan Gao, Lijuan Jiang, Lingli Tang

**Affiliations:** Department of Laboratory Medicine, The Second Xiangya Hospital of Central South University, Changsha, China

**Keywords:** STING agonist, STING inhibitor, bibliometric analysis, cancer immunotherapy, innate immune

## Abstract

**Background:**

STING is a core signaling hub molecule in the innate immune system, involved in various diseases, including infectious diseases, autoimmune diseases, tumors, aging, organ fibrosis, and neurodegenerative diseases. Its activation has shown great potential in anti-tumor and anti-infective therapies, with STING agonists emerging as a promising approach in cancer immunotherapy in recent years. This study identifies research trends and potential directions in the field by collecting and analyzing relevant literature.

**Methods:**

A total of 527 publications regarding STING agonists and 107 about inhibitors were retrieved from the WOS Core Collection database. Bibliometric information was extracted with CiteSpace and VOSviewer software for visualization.

**Results:**

It shows that research on both STING agonists and inhibitors is burgeoning rapidly. The United States and China are leading contributors in this field. Application of STING agonists primarily focuses on cancer immunotherapy, while STING inhibitors target inflammation, particularly neuroinflammation and acute lung injury.

**Conclusion:**

Current research emphasizes optimizing STING agonists for permeability, efficacy, and safety, with nanotechnology and lipid nanoparticles being prominent delivery techniques. Future research is expected to focus on drug development and clinical applications. This comprehensive bibliometric analysis provides clinical insights and a guide for further investigation to STING agonist/inhibitor.

## 1 Introduction

Stimulator of interferon genes (STING) was identified as a core adaptor protein in DNA sensing during 2008–2009 by four independent groups: Glen Barber, Hongbing Shu, Zhengfa Jiang, and John Cambier (Ishikawa and Barber, 2008; Jin et al., 2008; Zhong et al., 2008; Wenxiang et al., 2009). As a signaling hub in innate immunity, STING is a danger signal sensor in orchestrating the body’s response to pathogenic, tumor, or self-DNA in human diseases. In the classical STING signaling pathway, pathogen DNA, Cyclic Dinucleotides (CDNs) substances, tumor dsDNA, or aberrant cytosolic mtDNA are recognized by cyclic GMP-AMP (cGAMP) synthase (cGAS) (Decout et al., 2021), which then catalyzes the formation of cGAMP from GTP and ATP. cGAMP mobilizes STING conformational change and initiates the translocation of STING from the ER to the Golgi apparatus. During the translocation process, STING recruits TANK binding kinase 1 (TBK1) and interferon regulatory factor 3 (IRF3), which then translocate into the nucleus, inducing the expression of type I IFN (IFN-I) response, NF-kB activation, and other inflammatory cytokines, unleashing innate immune responses and establishing a ubiquitous and effective surveillance system against tissue damage and pathogen invasion (Chen and Xu, 2023). Activation of STING can induce a potent immune response against pathogen infections and cancer, while imbalances of this pathway may lead to a variety of human diseases, including infectious diseases, autoimmune diseases, tumors, organ fibrosis, and neurodegenerative diseases (Hopfner and Hornung, 2020; Dvorkin et al., 2024).

Over the past decade, the field of STING-related research has experienced rapid development, attracting widespread attention from numerous scholars. With the gradual elucidation of the cellular and pathophysiological mechanisms of cGAS-STING, the role of STING in many diseases has been clarified. Subsequently, new immunotherapeutic methods targeted to the regulation of STING have been developed, such as the use of STING agonists and inhibitors. STING agonists have been an emerging strategy for cancer immunotherapy (Minlin et al., 2020; Cai et al., 2023; Chen Y. et al., 2024; Wang J. et al., 2024). Through targeting antigen-presenting cells such as dendritic cells (DCs) and macrophages, STING agonists not only activate innate immune response but also sensitized and enhanced T-cell immunity, thus showing efficacious immune activation and antitumor effects in a spectrum of clinical and preclinical studies. In addition to antitumor treatment, STING agonists have also been applied in anti-infective treatment and vaccine development (Skouboe et al., 2018a; Humphries et al., 2021a; Zhang Y. et al., 2023; Liu et al., 2024). Mounting evidence has indicated that the abnormal activation of the cGAS-STING pathway is closely associated with the development of autoimmune diseases (AID) (Liu and Pu, 2023), nonalcoholic fatty liver disease (NAFLD), chronic inflammation, Parkinson’s disease, and cardiovascular diseases (Oduro et al., 2022). STING inhibitors also hold potential therapeutic value for these diseases (Shen et al., 2022; Liu and Pu, 2023).

Bibliometric analysis applies statistical methods and mathematical models to analyze bibliographic data, including publications, citations, and other scholarly communications. It is used to assess the productivity of researchers, institutions, and countries in a specific field (Anton et al., 2022). Additionally, it offers valuable insights for developing guidelines, making decisions, and treating diseases (Wilson et al., 2021). It is helpful to analyze the publication trends, disciplinary frontiers, hot contents, collaborative relationships, and influential factors of scholarly works (Romanelli et al., 2021). Bibliometric research can reveal the temporal dynamics of academic focus in a particular field, capture the hallmarks of emerging research areas, identify frontier issues in scholarly research, adjust the direction of scientific funding, and, more importantly, provide significant reference resources for literature reviews.

Therefore, conducting a bibliometric analysis of STING agonists/inhibitors is necessary to assess the current state of this emerging therapeutic and pharmaceutical strategy, explore the research scope and content within the field, and reasonably assume the future development direction. In this study, we performed a bibliometric analysis of STING agonists and antagonists, including publications from 2013 to 2024, to identify publication trends and highlight hotspot research areas.

## 2 Methods

### 2.1 Data collection and analysis

**FIGURE 1:**
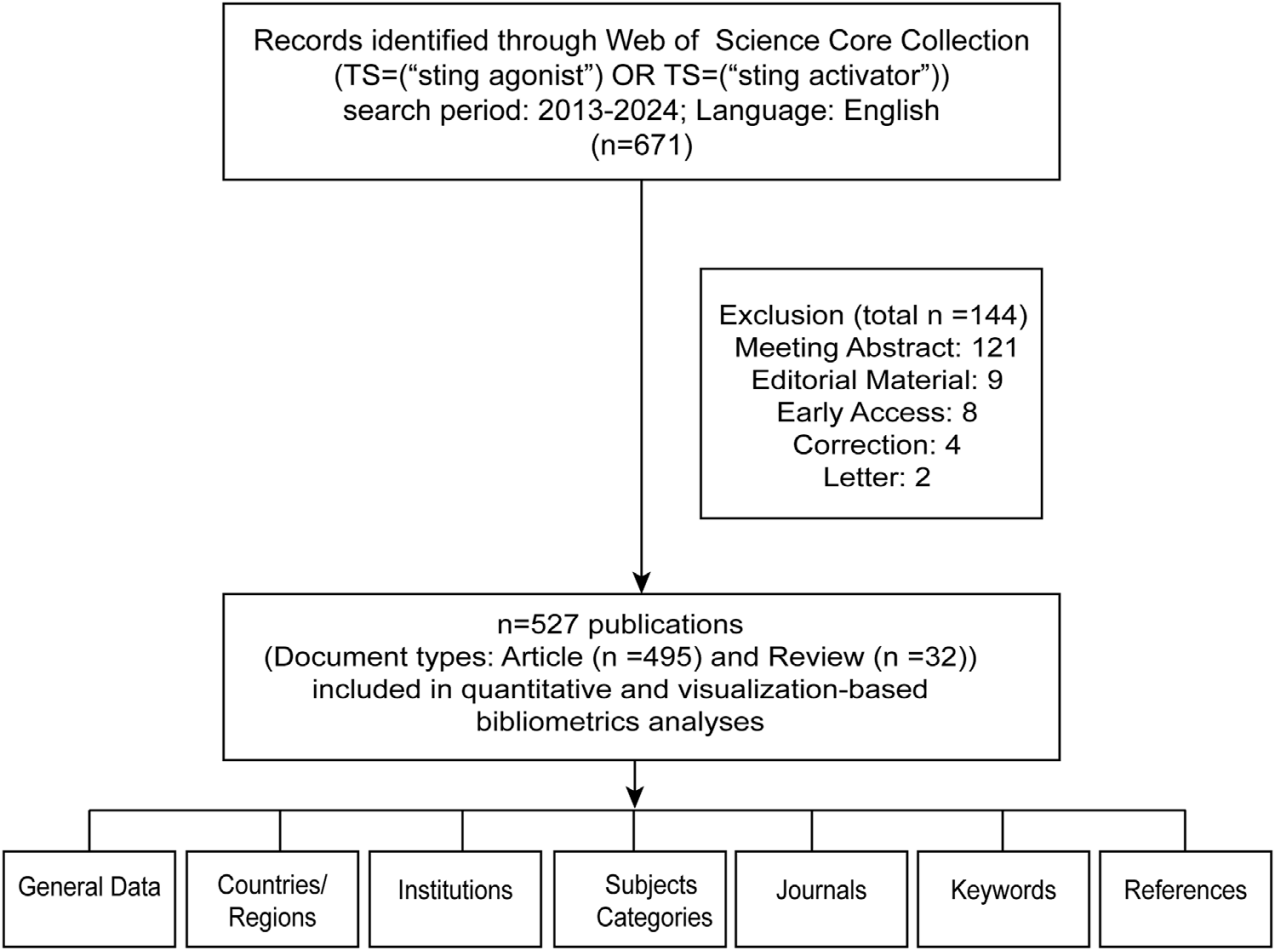
Flow chart of this study.

To achieve an exhaustive and accurate literature search, the Web of Science core collection database, which contains data from SCIE and SSCI, was queried using the following search terms on 11 August 2024: TS= (“sting agonist”) OR TS= (“sting activator”) for literature related to STING agonists; TS= (“sting inhibitor”) OR TS= (“sting antagonist”) for literature on STING inhibitors. The inclusion criteria for this study were as follows: English language only; document types limited to articles or review articles. Ultimately, 527 articles regarding STING agonists and 107 about STING inhibitors met the inclusion criteria and were retrieved from the WOS Core Collection database, as shown in Figure 1.

In this study, VOSviewer (version 1.6.20) and CiteSpace (version 5.8. R1.7z) were developed based on the JAVA programming language and have a robust visualization function. They were used to extract bibliometric information for co-cited references, countries, institutions, journals, co-authorship networks, keyword co-occurrence networks, and burstiness analyses and then generate bibliometric map visualization, calculation and analysis of co-occurrence network terms, national collaboration axis, co-citation, and bibliographic coupling of inter-author network relationships extracted from publication titles and abstracts. For descriptive analysis, the maximum, minimum, average, trend, and median were provided. To offer a more in-depth perspective on the translational potential of STING agonists and inhibitors, we incorporated a statistical analysis of the global drug development database for STING agonists and inhibitors extracted from the “Pharnextcloud” platform (https://data.pharnexcloud.com/).

### 2.2 Validity and reliability

All citation information was exported from the ISI WoS database in.txt format and subsequently imported into VOSviewer and CiteSpace. Two reviewers (QW and YG) independently reviewed these data searches to exclude irrelevant content and then reached a final agreement. In case of disagreement between the two researchers, a third researcher intervened to resolve the dispute.

## 3 Results

### 3.1 The annual trend of article publication quantity and journal analysis

**FIGURE 2:**
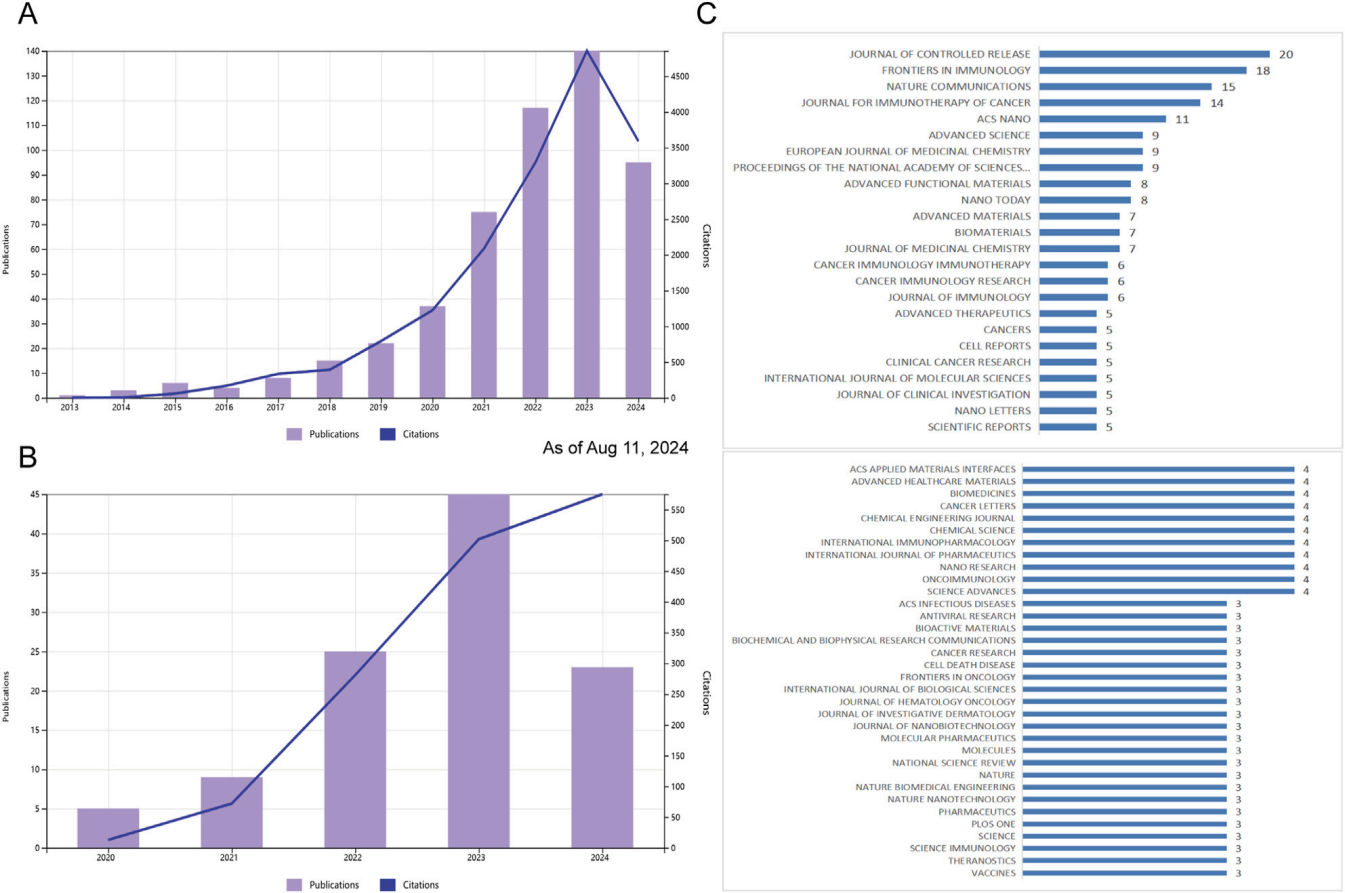
Publication and citation trends and journal distribution for STING agonist and inhibitor research (as of 11 Aug 2024). **(A)** Annual publication count and citation trends for STING agonist research from 2013 to 2024. **(B)** Annual publication count and citation trends for STING inhibitor research from 2013 to 2024. **(C)** Top journals publishing studies related to STING agonists and inhibitors, with counts indicating the number of relevant articles.

Publication numbers extracted from the WOS Core Collection database are classified by year. The evolution of STING agonist research is depicted in Figure 2A, while the research on STING inhibitors is presented in Figure 2B. Research on STING agonists has emerged as a focus over the past decade and has gained significant attention in recent years. In contrast, research on STING antagonists started later but has also shown a strong growth momentum in recent years. There are still many questions to be addressed and a great deal of space to explore regarding the regulation of STING and the application of its agonists/ antagonists. As the theoretical framework in this field continues to deepen and improve, the pace of publication will continue to grow rapidly in the next 5 years.

A total of 200 journals published the literature, as shown in Figure 2C. The top 10 journals [ranked by the number of publications (NPs)] are presented in Table 1 along with additional journal information, including the NPs, number of citations (NCs), journal impact factor (IF), average citations per item [also known as citations per article (CA)], and H-index. The JOURNAL OF CONTROLLED RELEASE published the highest number of relevant articles. CA serves as an indicator of contribution impact in a specific domain. A higher CA figure means a greater contribution. Although every journal did not publish similar articles, they played a significant role in shaping this sphere. In particular, NATURE COMMUNICATIONS (n = 15, CA = 49.6), JOURNAL FOR IMMUNOTHERAPY OF CANCER (n = 14, CA = 46.64), and the PROCEEDINGS OF THE NATIONAL ACADEMY OF SCIENCES OF THE United States (n = 9, CA = 33.56) showed a substantial impact. These findings indirectly highlight the significance of STING regulation and the potential application of STING agonists/antagonists.

**TABLE 1:** Quantitative measurement of top 10 journals publishing articles about STING agonists and/or inhibitors.

Journal	NP	NC	IF (2023)	Average citations per item	H-index
JOURNAL OF CONTROLLED RELEASE	20	345	10.5	17.6	10
FRONTIERS IN IMMUNOLOGY	18	133	5.7	7.5	7
NATURE COMMUNICATIONS	15	742	14.7	49.6	10
JOURNAL FOR IMMUNOTHERAPY OF CANCER	14	648	10.3	46.64	11
ACS NANO	11	178	15.8	16.36	7
ADVANCED SCIENCE	9	69	14.3	7.78	4
EUROPEAN JOURNAL OF MEDICINAL CHEMISTRY	9	64	6	8.11	4
PROCEEDINGS OF THE NATIONAL ACADEMY OF SCIENCES OF THE United States	9	302	9.4	33.56	8
ADVANCED FUNCTIONAL MATERIALS	8	191	18.5	24	5
NANO TODAY	8	73	13.2	9.25	2

NP, number of publications; NC, number of citations.

### 3.2 Publication distribution across WOS categories

**FIGURE 3:**
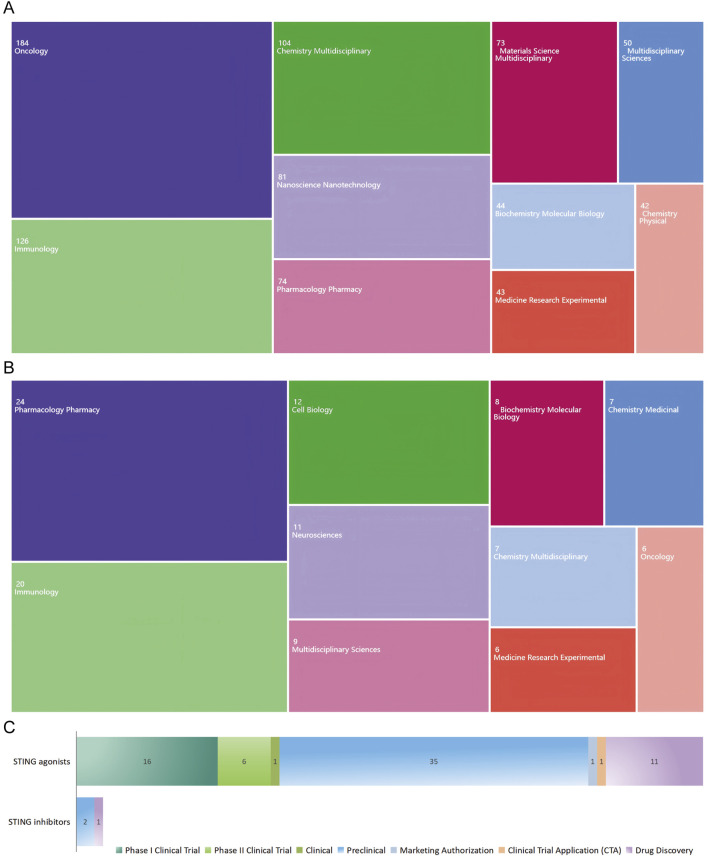
Publication distribution across WOS categories and drug development overview. **(A)** Publication categories related to STING agonists. **(B)** Publication categories related to STING inhibitors. **(C)** STING agonist and inhibitor development stage overview.

The top 10 article categories related to STING agonists are presented in Figure 3A. Over half of the publications were classified as “Oncology” (n = 184, 34.92%), followed by “Immunology” (n = 126, 23.91%), “Chemistry Multidisciplinary” (n = 104, 19.73%), “Nanotechnology” (n = 81, 15.37%), and “Pharmacology Pharmacy” (n = 74, 14.04%). As shown in Figure 3B, the top five article categories related to STING inhibitors are “Pharmacology Pharmacy,” “Immunology,” “Cell biology,” “Neuroscience,” and “Multidisciplinary science.” According to the categorization result, STING agonists are more advanced in clinical application, particularly in oncology. In contrast, STING inhibitors are in the early stages of research and development, with a broader therapeutic potential under exploration. For further understanding, we referred to the global drug development database for STING agonists and inhibitors from the “Pharnextcloud” platform (https://data.pharnexcloud.com/). The analysis, as depicted in Figure 3C, reveals that most STING agonists are in the preclinical and Phase I clinical trial stages. Some are in Phase II clinical trials, while there are very few STING inhibitors under development. Additional details, including company information, indications, and target molecules, are compiled in Supplementary Table S1. Additionally, we conducted a keyword timeline and clustering analysis of the publications from the top five categories, as well as counterparts in the field of pathogenology, as shown in Supplementary Figures S1–S6.

### 3.3 Publication distribution and collaborative network across countries, institutions, and authors

**FIGURE 4:**
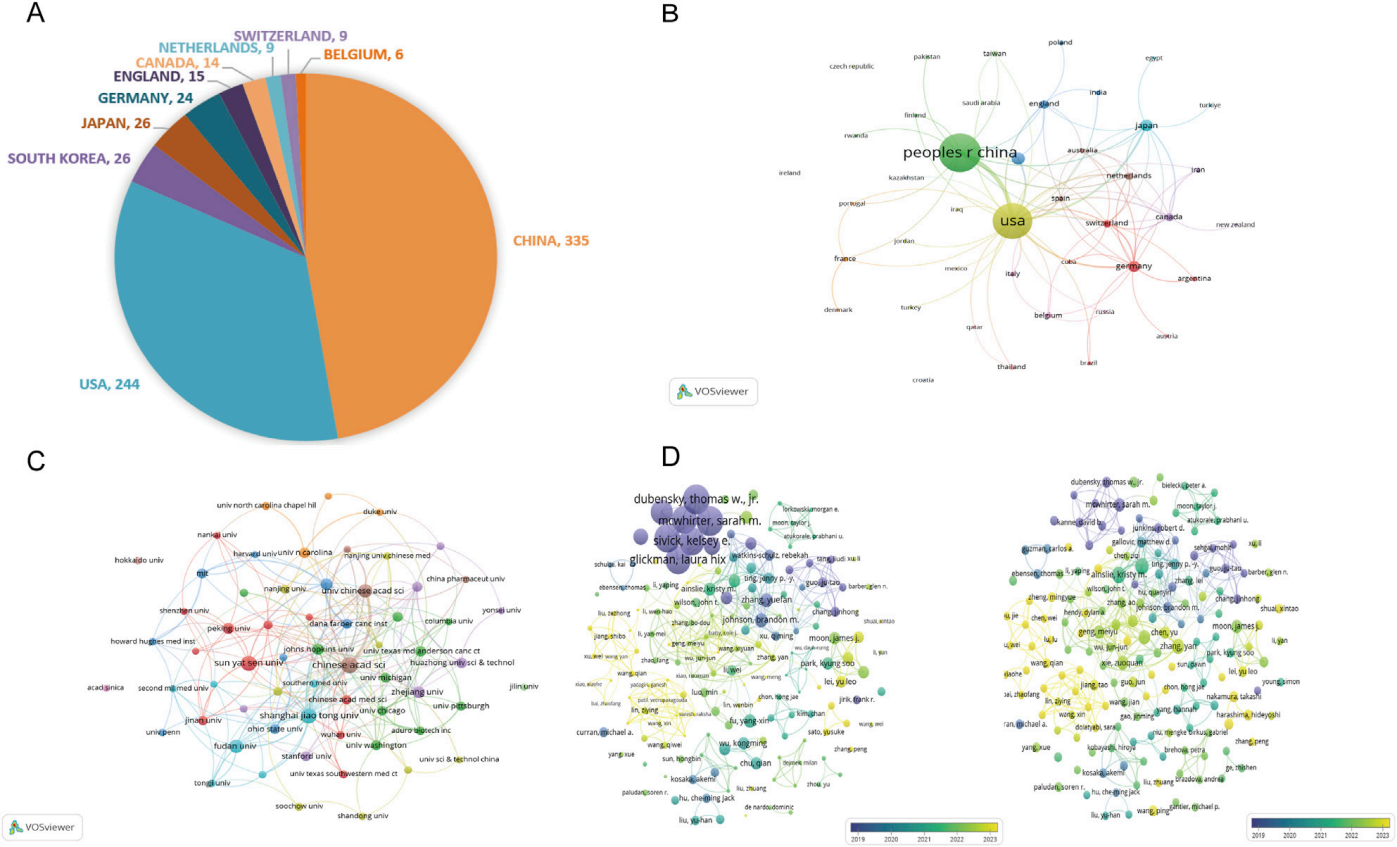
Global distribution and collaborative networks in STING agonist/inhibitor research. **(A)** The top 10 most productive countries according to the number of articles. **(B)** Visualization of international collaborative relationships. **(C)** Visualization of the institutional network. **(D)** Visualization of co-authorship network.

These articles were published across 42 countries. As shown in Figure 4A, the top 10 most productive countries are China (n = 335, 63.57%), the United States (n = 244, 46.30%), South Korea (n = 26, 4.93%), Japan (n = 26, 4.93%), and Germany (n = 24, 4.55%), followed by England (n = 15, 2.85%), Canada (n = 14, 2.65%), Netherlands (n = 9, 1.71%), Switzerland (n = 9, 1.71%), and Belgium (n = 6, 1.14%). The collaboration network was visualized by VOSviewer, as depicted in Figure 4B, which reflects international collaborations, with a higher number of lines indicating greater activity and the thickness of the line between two countries indicating the strength of the collaborative relationship. China has the most publications, while the United States has the highest collaborative strength. The United States and China had the most robust links.

The top 10 most prolific institutions are presented in Table 2, mirroring the county contribution ranking. They are all from China and the United States, and the CHINESE ACADEMY OF SCIENCES has published the most articles (n = 38, NC = 681, China, CA = 18.45, H = 15), while the UNIVERSITY OF TEXAS SYSTEM gained the highest number of citations (n = 27, NC = 1482, the United States, CA = 55.52, H = 17). The partnership visualization of each institution is presented in Figure 4C. Chinese universities have strong and close collaborative ties with one another, which may drive their high productivity.

**TABLE 2:** Quantitative measurement of top 10 institutions conducting STING agonist/inhibitor research.

Affiliations	NP	NC	Country	Average citations per item	H-index
CHINESE ACADEMY OF SCIENCES	38	681	China	18.45	15
UNIVERSITY OF TEXAS SYSTEM	27	1482	United States	55.52	17
HARVARD UNIVERSITY	24	1300	United States	54.5	15
SHANGHAI JIAO TONG UNIVERSITY	24	566	China	23.83	11
SUN YAT SEN UNIVERSITY	24	416	China	17.54	11
FUDAN UNIVERSITY	19	459	China	24.68	9
UNIVERSITY OF CHINESE ACADEMY OF SCIENCES CAS	19	218	China	12	9
HARVARD MEDICAL SCHOOL	17	892	United States	52.88	12
MASSACHUSETTS INSTITUTE OF TECHNOLOGY MIT	16	772	United States	48.75	11
UNIVERSITY OF NORTH CAROLINA	16	633	United States	41.19	9

NP, number of publications; NC, number of citations.

The co-authorship analysis of researchers is visualized in Figure 4D. Based on the volume of citations (as shown on the left), the collaborative team of Sarah M. McWhirter, Laura Hix Glickman, David B. Kanne, Thomas W. Dubensky, Jr., and Kelsey E. Sivick has published the most highly cited articles. In terms of the volume of documents (as shown on the right), Kristy Ainslie and Eric M. Bachelder have the highest NPs. Chinese scholars have published relatively newer literature, indicating their rapid exploration and application of new research directions. This highlights a solid scientific research foundation of the United States and China’s strength in applied innovation in this field. In general, the United States and China have made significantly far greater contributions to this field than other countries, and their responses to new research directions are more timely and rapid.

### 3.4 Analysis of keyword co-occurrence and bust

**FIGURE 5:**
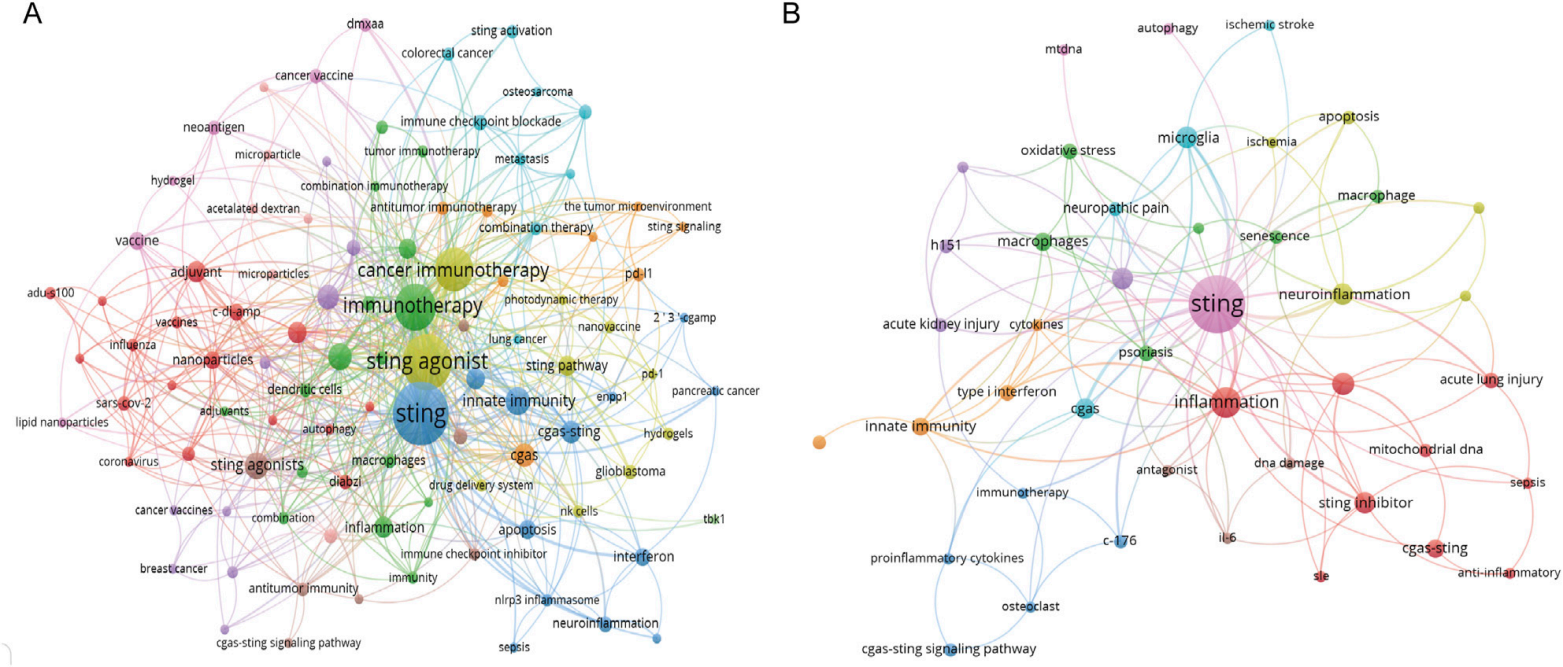
Analysis of the co-occurrence network of keywords. **(A)** Co-occurrence network of keywords related to STING agonists. **(B)** Co-occurrence network of keywords related to STING inhibitors.

Keywords were extracted from the titles and abstracts and then analyzed using VOSviewer and Citespace. Keyword analysis is helpful in monitoring scientific questions and highlighting emerging hotspots. In Figure 5, the significance of each term is highlighted by the size of the circle, with a larger circle indicating greater importance. Lines connecting the terms represent the association, and the thickness of the lines indicates the strength of the association between the two terms. By pointing to a specific keyword, one can obtain research directions and hotspots related to that keyword. Keywords co-occurrence analysis related to STING agonists is shown in Figure 5A, while STING antagonists are shown in Figure 5B. The research on STING agonists mainly centered around “cancer immunotherapy.” In addition, “adjuvant,” “innate immunity,” “nanoparticles,” “inflammation,” “apoptosis,” “immune checkpoint blockade,” and other aspects are also active research topics related to STING agonists in recent years. Inflammation is the main direction of exploration for STING antagonists, among which neuroinflammation and acute lung injury are important application directions.

A detailed display of keywords associated with the top four nodes is presented in Supplementary Figure S7. Among these hot words (minimum number of occurrences of a keyword is more than five; 45 words meet the threshold) that co-occur with STING agonist include: STING, cancer immunotherapy, immunotherapy, tumor microenvironment, innate immunity, colorectal cancer, macrophages, type I interferon, diABZI, photothermal therapy, immunogenic cell death, cGAS-STING pathway, combination therapy, SARS-CoV-2, adjuvant, c-di-AMP, cGAS, cyclic dinucleotides, cGAMP, nanoparticles, melanoma, glioblastoma, DMXAA, cancer, antitumor immunity, vaccine, neoantigen, and drug delivery. For the specific STING agonists and inhibitors, Table 3 summarizes the keywords co-occurring with specific STING agonist drugs, such as c-di-AMP, 2′3′-cGAMP, DMXAA, ADU-S100, 2′3′-c-di-AM(PS)2 (Rp,Rp), and diABZI, MSA-2, and with specific STING inhibitor drugs, such as C176, H151, ISD-017, as well as their usage and main producers.

**TABLE 3:** Information and co-occurrence keywords of specific STING agonists and inhibitors.

Categories	Drugs	Usage	Producers	Keywords co-occurrence
STING agonists	c-di-AMP	i.p. (Skouboe et al., 2018b)	InvivoGen	Hepatocellular carcinoma, Radiotherapy, Autophagy, Immunomodulation, Sars-cov-2, Adjuvant, Vaccine, Antigen delivery, Innate immune response, Inflammasome, Mucosal adjuvant, Mucosal vaccine, BCG, Gut microbiome, NOD-like receptor with pyrin domain (Nalt), Trypanosoma cruzi, Trans-sialidase, Dec-205, Subcutaneous route, Archaeosomems, HCV, STING agonist
	2′3′-cGAMP	i.t. (Sibal et al., 2024) i.p. (Skouboe et al., 2018b) i.n. (Shao et al., 2023; Leekha et al., 2024) intrathecally (Donnelly et al., 2021)	InvivoGen MCE	Photothermotherapy, Pancreatic cancer, Oncolytic virus, Cell transmission, Herpes simplex virus type 1(HSV-1), ENPP1 gene, Chloroquine, Innate immunity, Cancer vaccine, Antitumor effect, TME, STING agonist
	DMXAA (Vadimezan)	i.t. (Ding et al., 2020) i.p. (Skouboe et al., 2018b) intrathecally (Donnelly et al., 2021)	InvivoGe MCE Selleckchem	Innate immunity, Immunotherapy, Combination therapy, Metastasis, Immune checkpoint blockade, Triple-negative breast cancer, and Derivative
	ADU-S100	i.t. (Ding et al., 2020) intrathecally (Donnelly et al., 2021)	InvivoGen	Vaccination, Cellular immunity, Nano-11, Pigs, Swine influenza A viruses (SIVs), Swine, Intranasal vaccination, Memory responses, Cell-mediated immune responses, Retinoic acid-related orphan receptor gamma T (ROR-γt), 2′3′-c-di-AM(PS)2 (Rp,Rp), DCs, and Cancer immunotherapy
	2′3′-c-di-AM(PS)2 (Rp,Rp)	NA	InvivoGen	ADU-S100, Human Th-17 cells, ROR-γt, Glioblastoma, STING agonist
	diABZI	i.v. (Jm et al., 2018) i.t. (Chen et al., 2024a) i.p. (Humphries et al., 2021a) i.n. (Humphries et al., 2021b) intratracheally (Messaoud-Nacer et al., 2022)	InvivoGen MCE Caymanchem Selleckchem Probechem	Autophagy, Apoptosis, Air-liquid interface clusters, Coronavirus, Sars-cov-2, Nonsmall cell lung cancer (NSCLC), TLR7/8 agonist, Mouse models, Psoriasis, and Macrophage
	MSA-2	i.t. and i.v. (Yang et al., 2024) s.c. and po. (Pan et al., 2020)	InvivoGen MCE	Manganese, Antiviral effect, Drug delivery, and Cancer immunotherapy
STING inhibitors	C176	i.p. (Shamseddine et al., 2023)	MCE Tocrisbio	Neuroinflammation, Inflammatory osteolysis, Apical periodontitis, Osteoclast differentiation, Osteoclast, Parkinson’s disease, Immunotherapy, and NLRP3 inflammasome
	H-151	i.p. (Shamseddine et al., 2023)	Invivogen Tocrisbio Caymanchem Selleckchem	Inflammation, Intestinal ischemia, Cisplatin, Acute kidney injury, Ischemia reperfusion, Myocardial infarction, Myocardial fibrosis, Mitochondria dysfunction, and Macrophage
	ISD-017	i.p. (Alee et al., 2024)	NA	Systemic lupus erythematosus (SLE), Lupus nephritis

i.p., intraperitoneal injection; s.c., subcutaneous injection; i.n., intranasal administration; i.t., intratumoral injection/administration; i.v., intravenous injection; po., oral administration; MCE, MedChemExpress.

**FIGURE 6:**
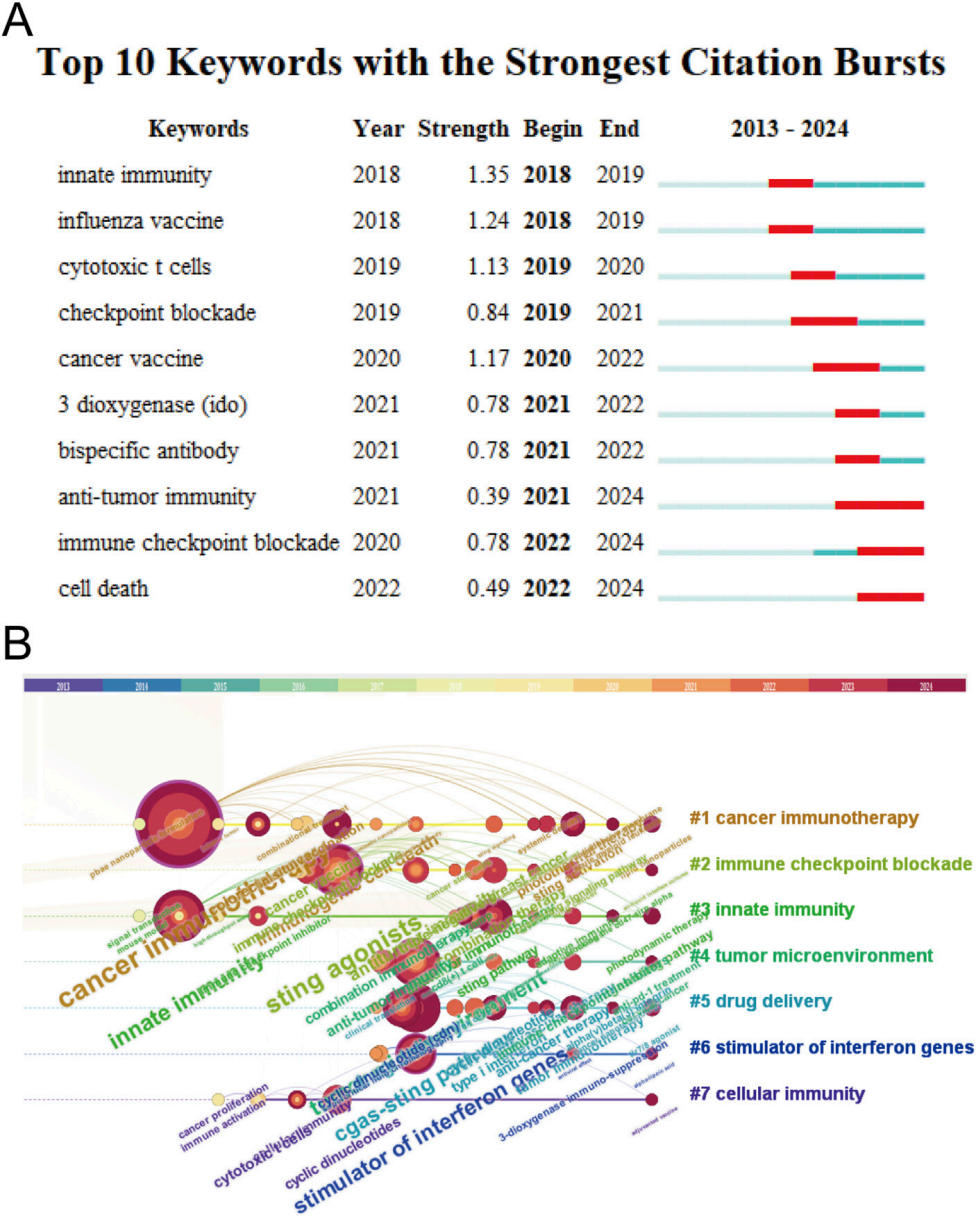
Citation bursts and conceptual networks from keyword analysis. **(A)** Top 10 keywords with the strongest citation bursts from 2013 to 2024. **(B)** Timeline view of research clusters based on keyword co-occurrence.

Through analysis with CiteSpace, it is able to identify research hotspots, reveal research trends, and predict future research directions. Keyword burst refers to a sudden and significant increase in citation frequency within a certain period of time. The citation burst of these keywords usually indicates research hotspots in the field. The red mark on the keyword timelines in Figure 6A indicates the importance of and attention to keywords in the research field: the longer the length, the longer the duration of keywords, and the stronger the research frontier. The top 10 keywords with the strongest citation bursts include innate immunity, influenza vaccine, cytotoxic T cells, checkpoint blockade, 3 dioxygenase (IDO), bispecific antibody, antitumor immunity, immune checkpoint blockade, and cell death, sorted by time. The timeline distribution and clustering of these keywords are shown in Figure 6B. A time slice is located at the top of the figure. The center color of the circle represents the time when a keyword first appeared, and the dark red of the circle indicates that the keyword continues to be a research hotspot to the present day. The larger the circle, the longer the research. Keywords with the same color font belong to the same cluster. Cancer immunotherapy and nanoparticle formulation are long-term research focuses. Drug delivery and immunology research are hot topics in recent years and may be future research directions.

### 3.5 Top 10 most-cited publications

**FIGURE 7:**
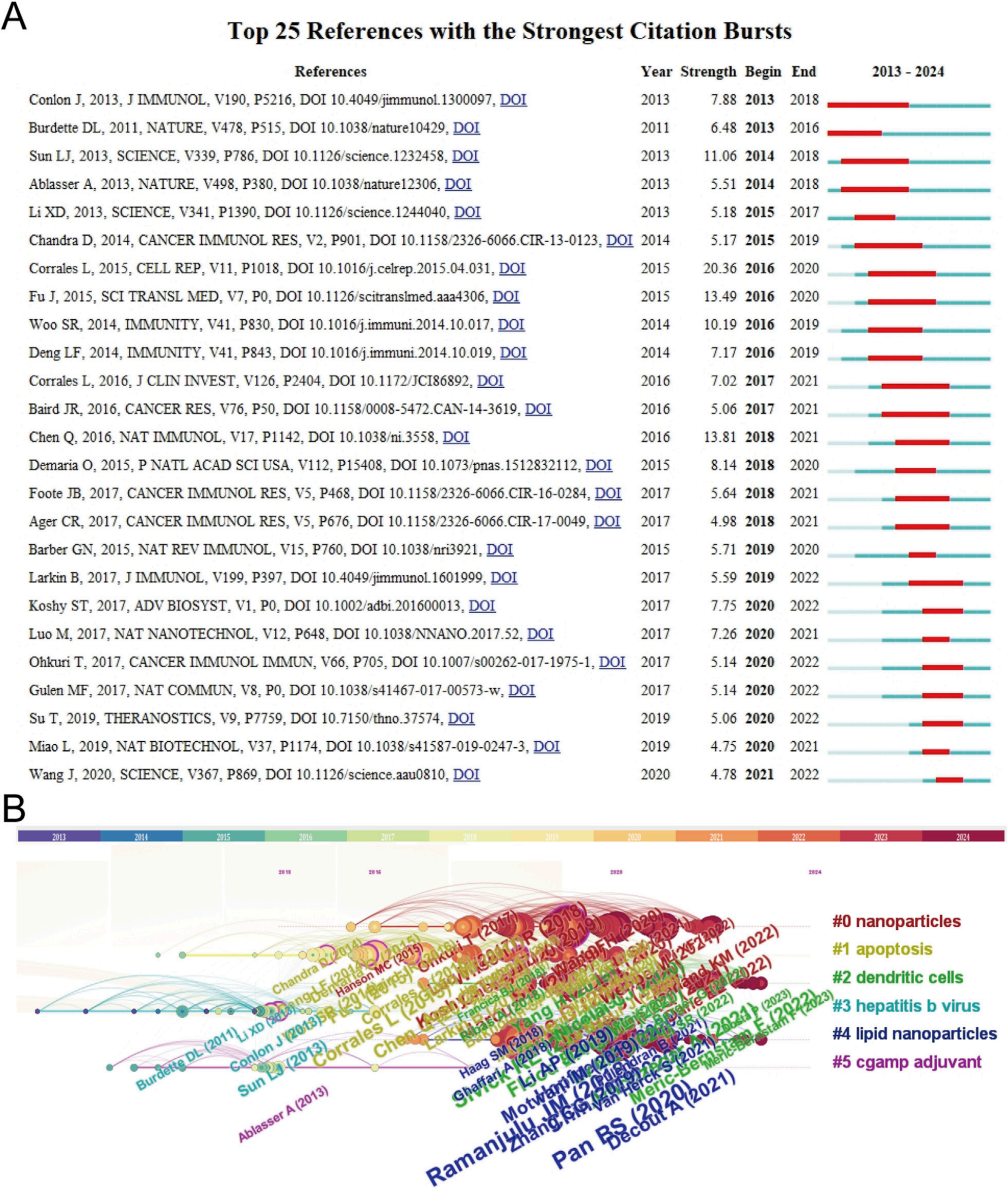
Citation bursts and conceptual networks from reference analysis. **(A)** Top 25 references with the strongest citation bursts. **(B)** Timeline view of thematic research topics based on references.

The top 10 most-cited papers about STING agonists are shown in Table 4. These publications were all published in prestigious journals between 2015 and 2021. All of them received a high number of citations per year. The top 25 references with the strongest citation bursts are shown in Figure 7A. Conceptual clusters were created when a set of publications were cited repeatedly together. A timeline view of the top five clusters divided by CiteSpace, nanoparticles, apoptosis, dendritic cells, hepatitis B virus, lipid nanoparticles, and cGAMP adjuvants is presented in Figure 7B. Publications about nanoparticles had the most citation bursts. Dendritic cells are the most extensively studied immune cells.

**TABLE 4:** Top 10 most-cited publications.

Articles	Author (year)	Source title	Citations/year	Citations
Direct activation of STING in the tumor microenvironment leads to potent and systemic tumor regression and immunity	Corrales, L (2015)	CELL REPORTS	99.8	998
STING agonist formulated cancer vaccines can cure established tumors resistant to PD-1 blockade	Fu, J (2015)	SCIENCE TRANSLATIONAL MEDICINE	53.7	537
Design of amidobenzimidazole STING receptor agonists with systemic activity	Ramanjulu, JM (2018)	NATURE	67.86	475
The cytosolic sensor cGAS detects *Mycobacterium tuberculosis* DNA to induce type I interferons and activate autophagy	Watson, RO (2015)	CELL HOST and MICROBE	44.5	445
Hydrolysis of 2′3′-cGAMP by ENPP1 and design of nonhydrolyzable analogs	Li, LY (2014)	NATURE CHEMICAL BIOLOGY	29.18	321
An orally available non-nucleotide STING agonist with antitumor activity	Pan, BS (2020)	SCIENCE	61.6	308
Amplifying STING activation by cyclic dinucleotide-manganese particles for local and systemic cancer metalloimmunotherapy	Sun, XQ (2021)	NATURE NANOTECHNOLOGY	73.75	295
Antitumor activity of a systemic STING-activating non-nucleotide cGAMP mimetic	Chin, EN (2020)	SCIENCE	50.6	253
Magnitude of therapeutic STING activation determines CD8^+^ T cell-mediated antitumor immunity	Sivick, KE (2018)	CELL REPORTS	35.71	250
STING-mediated inflammation in Kupffer cells contributes to progression of nonalcoholic steatohepatitis	Yu, YS (2019)	JOURNAL OF CLINICAL INVESTIGATION	39.67	238

NP, number of publications; NC, number of citations.

## 4 Discussion

### 4.1 Overview

Here, we clarified the current pattern of development trends and research hotspots in the field of STING agonist/antagonist literature. We have included all articles related to STING agonists and antagonists for journal analysis and analysis of collaborative networks across countries, institutions, and authors. Because STING is an emerging therapeutic target, research and development (R&D) efforts on both agonists and antagonists have shown soaring growth over the past decade, as indicated by rising annual publication figures and citation counts. Moreover, the top 10 journals by publication count and co-cited journals are all categorized as Q1, indicating their high quality and impact on the STING research field.

China and the United States are the leading contributors in the field. Both countries dominated the top 10 most productive institutions. Chinese institutions have established a strong collaborative network. The Chinese Academy of Sciences, the University of Texas System, Harvard University, Shanghai Jiao Tong University, and Sun Yat Sen University are the prominent contributors. This leadership can be attributed to a solid scientific research foundation of the United States and China’s strength in applied innovation in this field, as well as substantial investments. China also shows the most achievements in the study of the STING signaling pathway, establishing a perfect theoretical knowledge system.

Kristy Ainslie and Eric M. Bachelder (University of North Carolina at Chapel Hill), Zuoquan Xie (Chinese Academy of Sciences), Jenny P. Ting (University of North Carolina at Chapel Hill), and James J. Moon (University of Michigan) are the top five most prolific authors. The research collaboration team of Kristy Ainslie and Eric M. Bachelder focuses on the development of new vaccines and immunotherapies, including nanoparticle delivery systems for STING agonists and the application of STING agonists in seasonal influenza vaccines and autoimmune diseases. Their latest research showed that the encapsulation of cGAMP in acetylated dextran (Ace-DEX) microparticles (MPs) with multi-COBRA hemagglutinin enhances its intracellular delivery, eliciting protective immune responses against human seasonal and pre-pandemic strains (Hendy et al., 2024b; Zhang et al., 2024).

Xie primarily specializes in antitumor immunity, and his latest research reported a new compound 202 (C202) that made a structure modification to MSA-2 by replacing the carbonyl group with a difluoromethylene, exhibiting better oral bioavailability and plasma exposure than MSA-2. Encouragingly, it induced complete tumor regression in many tumor models of mice and stimulated powerful immune memory effects, contributing to long-term immune response against cancer (Wang et al., 2023; Ding et al., 2023). Furthermore, his team designed a new diABZI-based STING agonist, HB3089, currently undergoing preclinical development as a promising drug candidate that triggers substantial antitumor immunity and exhibits robust antitumor activity (Xie et al., 2022). Jenny P. Ting also specializes in developing a STING agonist delivery platform, new STING agonist compounds, and influenza vaccines (Watkins-Schulz et al., 2019; Gallovic et al., 2022; Yang et al., 2022). She recently reported a universal STING mimic (uniSTING) based on a polymeric architecture. It can selectively stimulate tumor control IRF3/IFN-I pathways but not tumor progression NF-κB pathways. Intratumoral or systemic injection of uniSTING-mRNA via lipid nanoparticles (LNPs) results in potent antitumor efficacy across triple-negative breast cancer, lung cancer, melanoma, and orthotopic/metastatic liver malignancies (Wang Y. et al., 2024).

James J. Moon has expertise in nanotechnology, immunotherapies, and combination treatments for cancer (Sun et al., 2020). He recently demonstrated a prototype of cancer metalloimmunotherapy by using manganese (Mn2+) that has been proven to effectively activate the cGAS-STING signaling pathway (Lv et al., 2020; Zhao et al., 2020; Sun et al., 2021). By focusing on the research contents of these outstanding scholars, we can keep abreast of the cutting-edge achievements in this field.

### 4.2 Research hotspots and future trends

We conducted individual keyword analysis and category analysis for STING agonists and antagonists. The application of STING agonists mainly focuses on cancer immunotherapy and vaccine adjuvants, as well as inflammation-associated diseases. Unlike agonists, STING inhibitors are being developed to mitigate unwanted STING activation, which is associated with chronic inflammatory diseases like lupus, Aicardi–Goutières syndrome, myocardial infarction, and other interferonopathies (Zhang et al., 2022). By inhibiting the pathway, researchers aim to reduce inappropriate immune responses triggered by self-DNA or chronic cytoplasmic DNA presence, which could otherwise lead to tissue damage and persistent inflammation. Neuroinflammation and AID treatment may be an important paradigm for the application of STING inhibitors.

According to the keyword co-occurrence and burst analysis, research in this domain primarily focuses on the following aspects:

(1) Development of new STING delivery vehicles: Scientists are employing a variety of novel technologies to modify STING agonists, aiming to synthesize ones with more targeted effects. STING agonists administered systemically often suffer from issues such as insufficient tumor accumulation, rapid clearance, and short duration of action, leading to limited therapeutic effectiveness (Van Herck et al., 2021; Li et al., 2023). To address these limitations, researchers are developing efficient agonist delivery platforms, including nanoparticle systems (Chen et al., 2023), lipid microparticles (Lu et al., 2020; Hao et al., 2023; Hendy et al., 2024a), ultrasound-activated vehicles (Jiang et al., 2023), and various polymers and bio-degradable materials that can encapsulate STING agonists (Huang et al., 2023). Thus, research in this area is multidisciplinary.

(2) Combined therapeutic strategies: To enhance the effectiveness of immunotherapy, researchers are combining nano-engineered STING agonists with other treatment modalities, such as immune checkpoint inhibitors (Su et al., 2019; Zhang P. et al., 2023), mild-temperature photothermal therapy (Ma et al., 2023), synergy with chemotherapy, combination with CAR-T therapy (Xu et al., 2021), and sono-activatable immunotherapy (Jiang et al., 2023). The primary strategy is to target and influence the tumor microenvironment (TME) to increase STING agonist accumulation within tumor tissues and amplify immune cell activation. The preliminary data showed that some combination therapies had encouraging antitumor activity with a tolerable safety profile (Yi et al., 2022).

(3) Exploring therapeutic effects across various diseases: The therapeutic impact of STING activation is complex. Although it has shown significant results in antitumor and antiviral applications, potential side effects such as cytokine storms and autoimmune diseases remain limiting factors. In striking contrast to their tumor suppressive roles, cGAS and STING have also been implicated in promoting tumor burden and worse disease outcomes in models of cancer (Decout et al., 2021). Additionally, studies on STING agonists in bacterial or fungal infections are limited. There is growing interest in using STING agonists for bacterial infections and chronic inflammatory diseases. The effects of STING agonists are being explored in various diseases, including melanoma, colorectal cancer, anti-vascular therapies, metastasis, pancreatic cancer, influenza, aging, breast cancer, cervical cancer, SARS-CoV-2, HIV, and *Mycobacterium tuberculosis* infections.

(4) Application as vaccine adjuvants (Tian et al., 2024): STING agonists can be applied in the development of cancer vaccines, including those for melanoma, breast cancer, lung cancer, and colorectal cancer, by activating the STING pathway to promote tumor antigen recognition and presentation, thus enhancing T cell-specific immune responses against cancer cells. Research on COVID-19 vaccines has further driven interest in STING agonists as vaccine adjuvants as they can enhance immune responses to SARS-CoV-2 and provide broad and durable protective effects (Humphries et al., 2021a; Zhang Y. et al., 2023). STING agonists also show potential in influenza (Luo et al., 2019), BCG (Erik et al., 2018), and HIV vaccines (Chattopadhyay and Hu, 2020) by promoting dendritic cell (DC) activation, increasing antibody production, and enhancing T-cell responses. As adjuvants in cancer and viral vaccines, STING agonists exhibit significant potential, with future research aiming to optimize their safety, efficacy, and delivery methods.

In summary, for STING agonists, the most urgent challenges include the development of drug delivery and release systems and understanding their working patterns *in vivo*, devising effective combination therapy strategies, and the development of biomarkers. When considering the developmental stages of cancer, the regulation of STING becomes complex. Some reports have also confirmed that chronic stimulation of STING can mediate tumor formation and metastasis through inflammation (Nambiar et al., 2023); thus, understanding their working patterns *in vivo* is important.

Combination therapy strategies address the resistance and inefficacy associated with the monotherapeutic use of STING agonists (Li et al., 2022). Biomarkers for monitoring the safety and efficacy of STING agonists/inhibitors therapy must be studied. Additionally, it is crucial for researchers across various fields to delve into the mechanisms by which STING operates in diseases, especially in inflammatory pathology during the course of disease. More clinical data need to be carefully analyzed and evaluated. STING inhibitors show promising prospects in the treatment of autoimmune diseases and neurodegenerative diseases. However, as small-molecule drugs, they also face the tricky issue of drug delivery system development. Their development is relatively recent and currently still in the preclinical or drug discovery stages. Understanding the dual role of STING signaling in different disease states may promote the development of STING inhibitors.

## 5 Conclusion

This analysis reveals the development trends in the research of both STING agonists and inhibitors, providing a detailed overview of this field. STING agonists show great potential in cancer immunotherapy, with a focus on enhancing their stability, cell permeability, and target concentration through advanced drug delivery systems like nanotechnology and lipid nanoparticles. Future research directions for STING agonists include developing high specificity and potency STING agonists, devising effective combination therapy strategies, and developing biomarkers. In contrast, STING inhibitors are emerging as promising candidates for the treatment of autoimmune and neurodegenerative diseases, facing the same challenges of drug delivery and specificity. Additionally, there is a growing interest in understanding the role of the cGAS-STING pathway in non-neoplastic diseases, which may reveal new opportunities for the application of both STING agonists and inhibitors.

## Data Availability

The original contributions presented in the study are included in the article/Supplementary Material; further inquiries can be directed to the corresponding author.
