# Supplementary material for: Profile of STING agonist and inhibitor research: a bibliometric analysis
Source: Front Pharmacol. 2025 Feb 11;16:1528459. doi: 10.3389/fphar.2025.1528459 (PMC11850258; doi:10.3389/fphar.2025.1528459)
Supplement: Supplementary file 2 [file Image1.pdf]

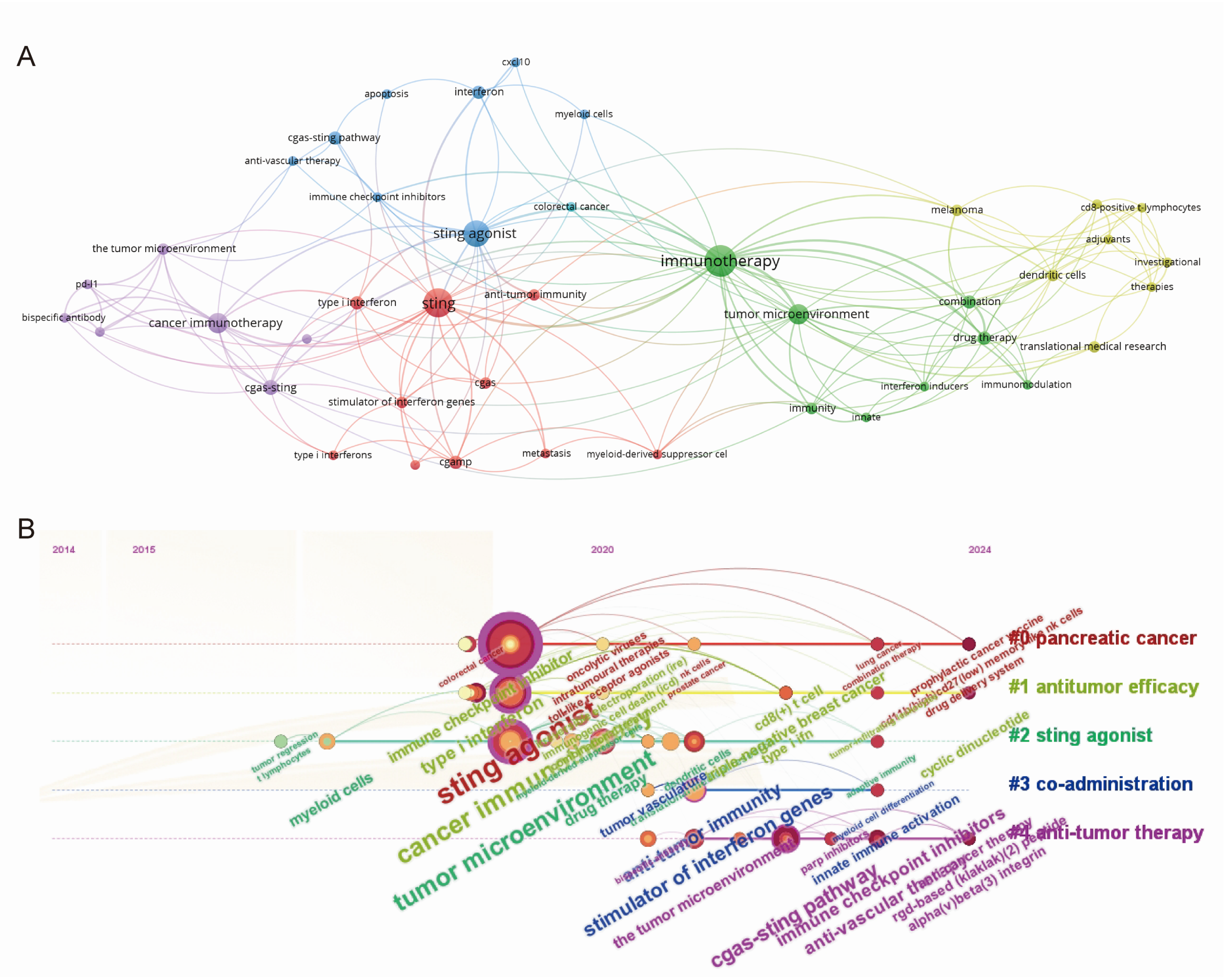

Supplementary Figure 1 (A) Co-occurrence network of keywords related to STING agonists, and (B) Timeline view of research clusters based on keyword co-occurrence in the field of onclogy.

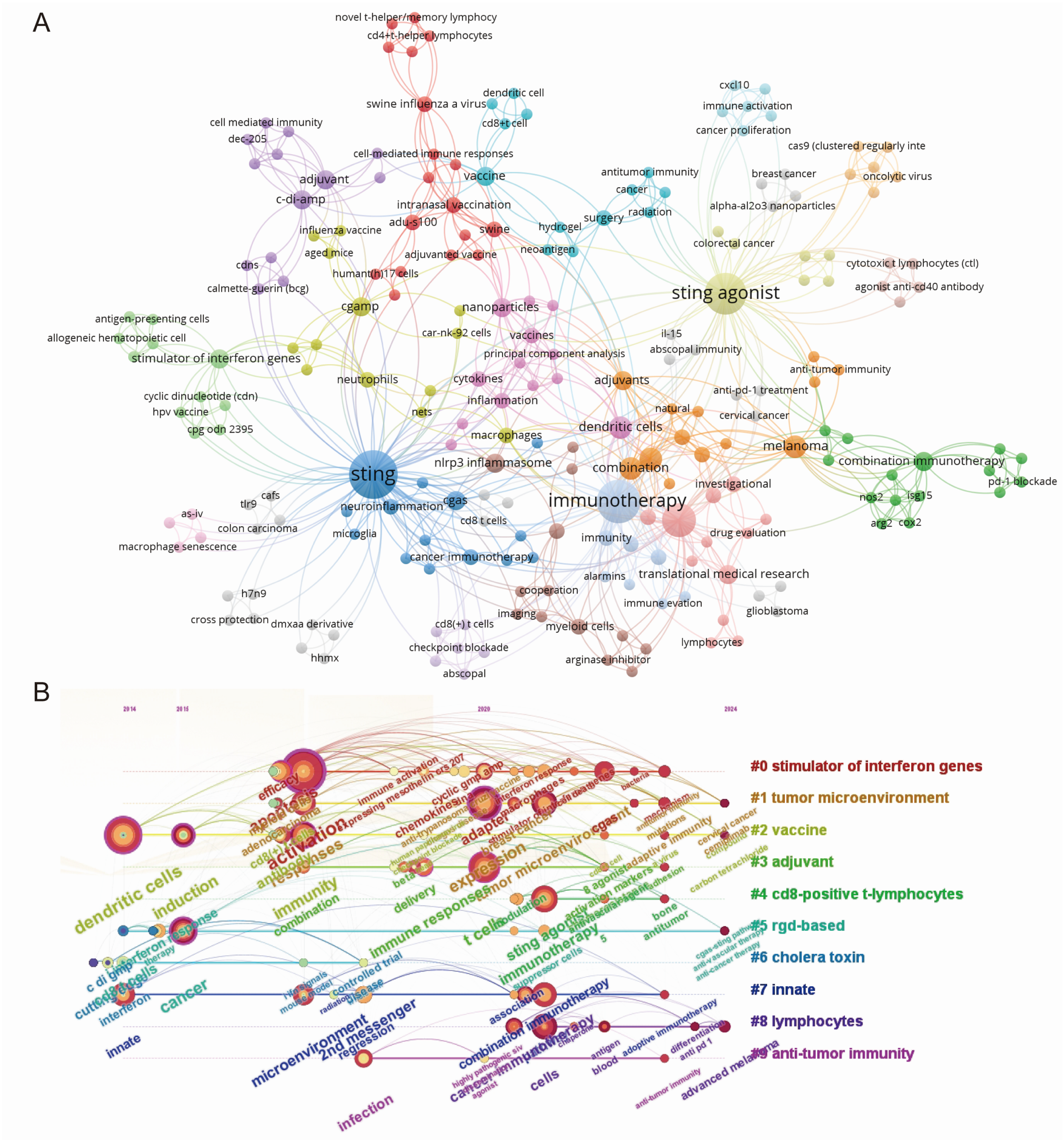

Supplementary Figure 2 (A) Co-occurrence network of keywords related to STING agonists, and (B) Timeline view of research clusters based on keyword co-occurrence in the field of immunology.

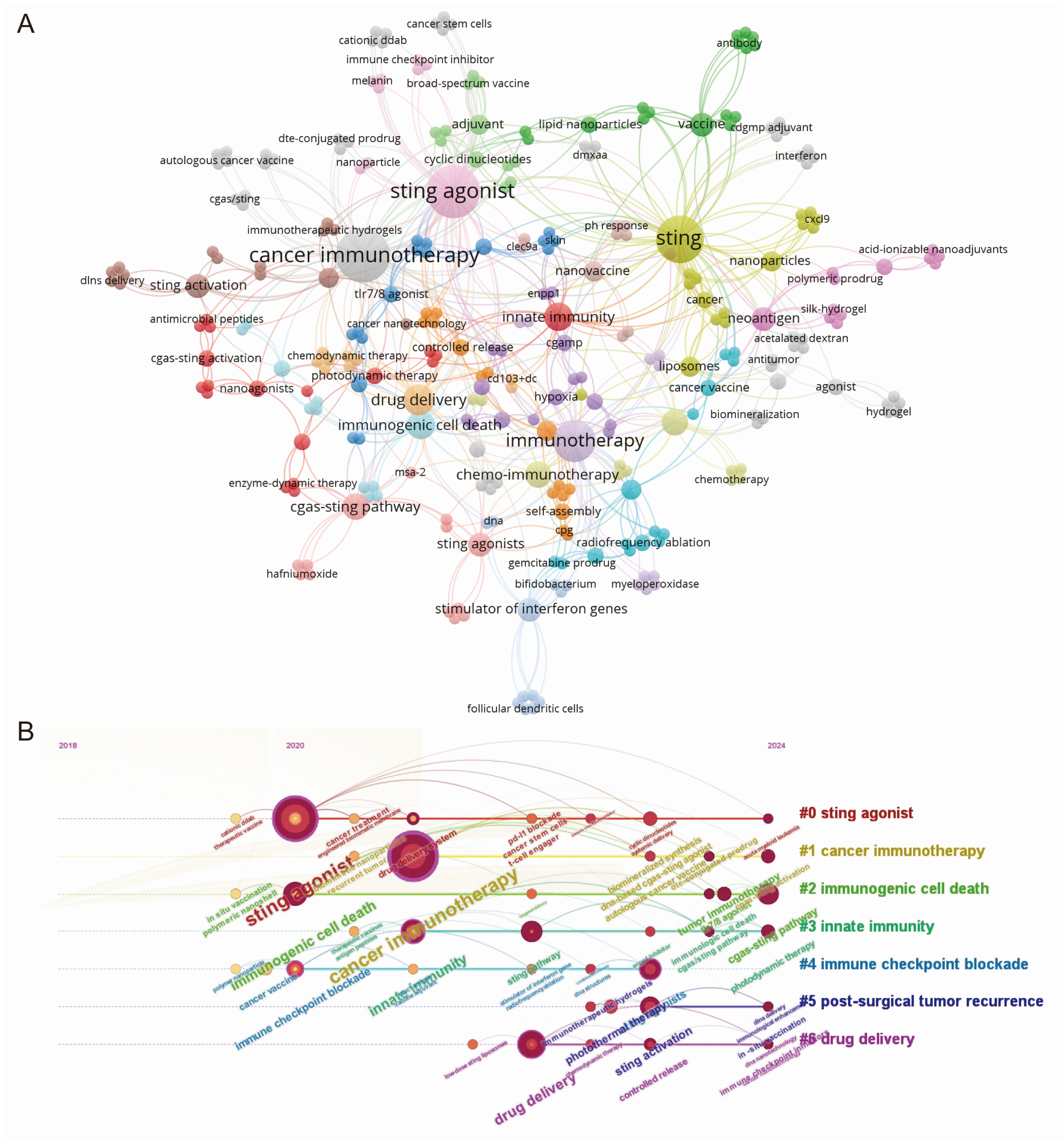

Supplementary Figure 3 (A) Co-occurrence network of keywords related to STING agonists, and (B) Timeline view of research clusters based on keyword co-occurrence in the field of chemistry multidisciplinary.

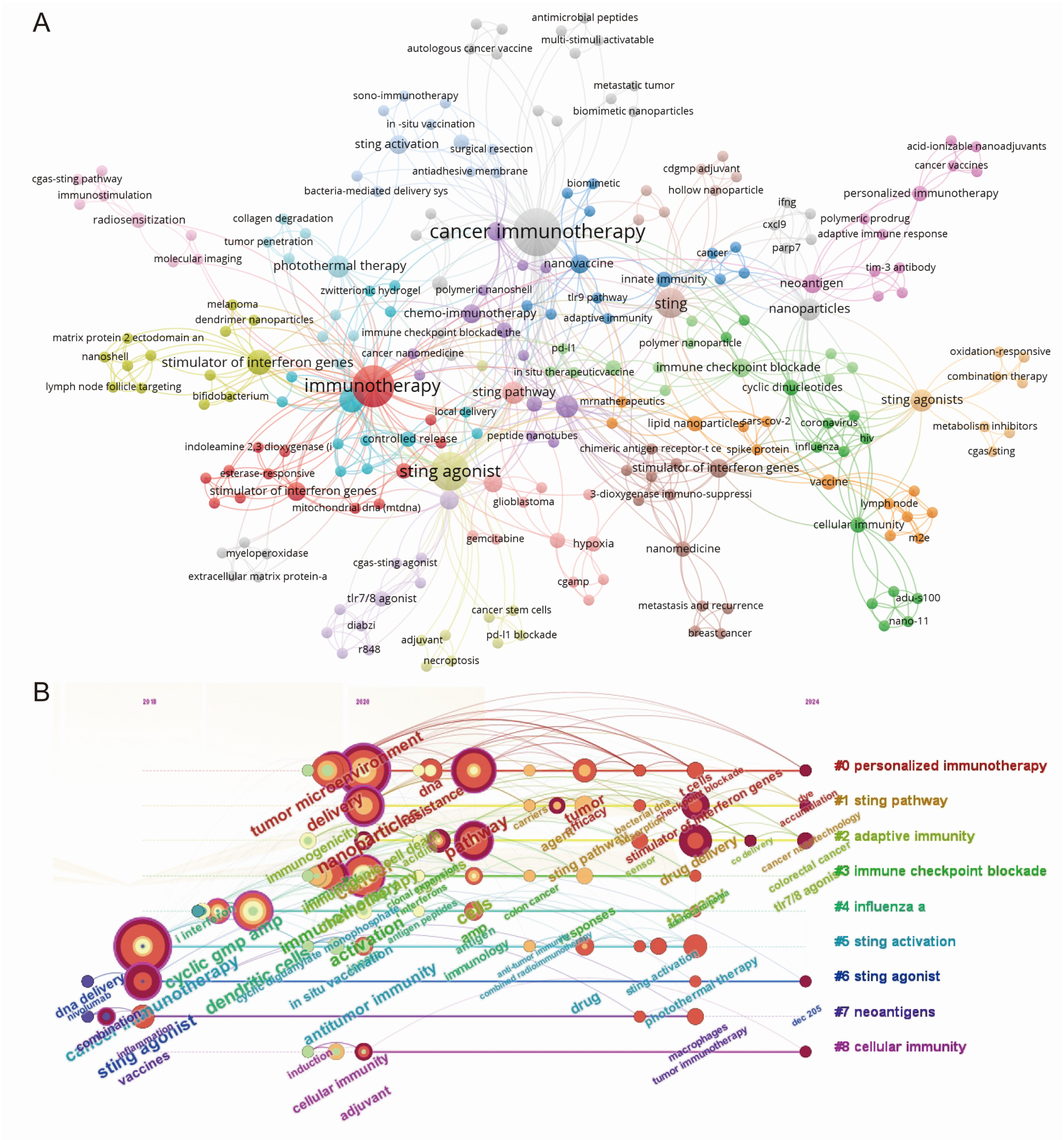

Supplementary Figure 4 (A) Co-occurrence network of keywords related to STING agonists, and (B) Timeline view of research clusters based on keyword co-occurrence in the field of nanotechnology.

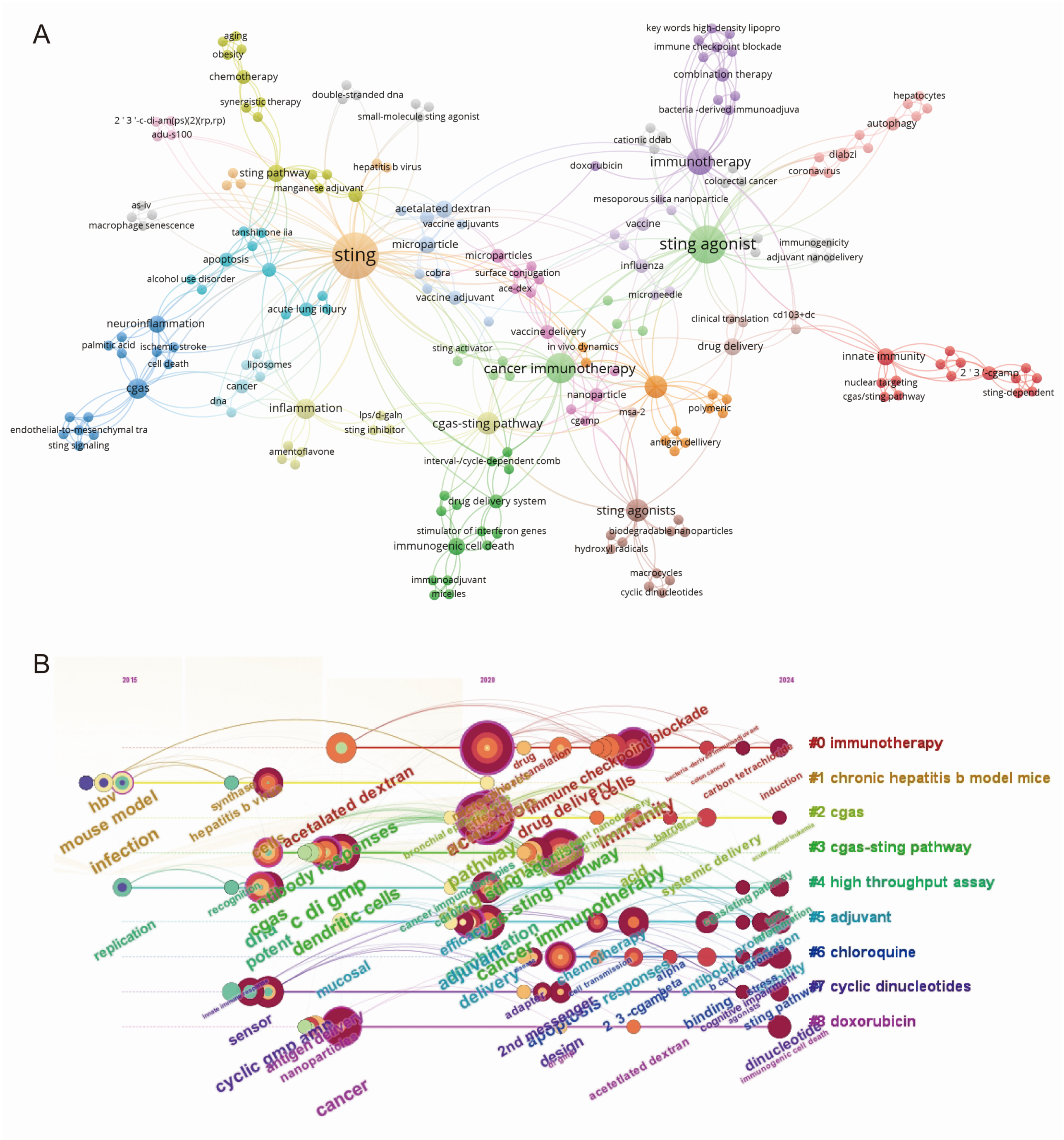

Supplementary Figure 5 (A) Co-occurrence network of keywords related to STING agonists, and (B) Timeline view of research clusters based on keyword co-occurrence in the field of pharmacology pharmacy.

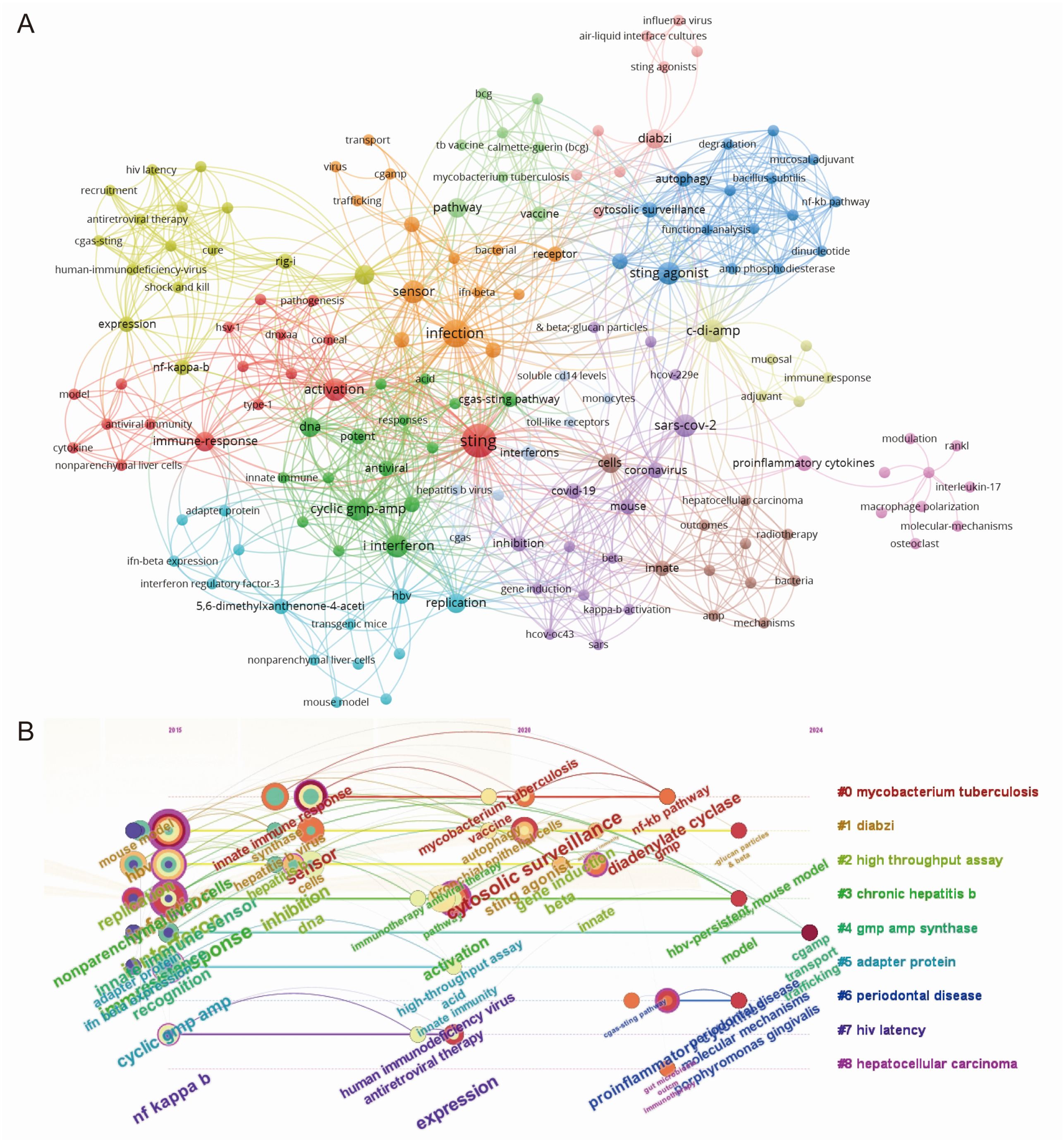

Supplementary Figure 6 (A) Co-occurrence network of keywords related to STING agonists, and (B) Timeline view of research clusters based on keyword co-occurrence in the field of virus and bacterium infection.

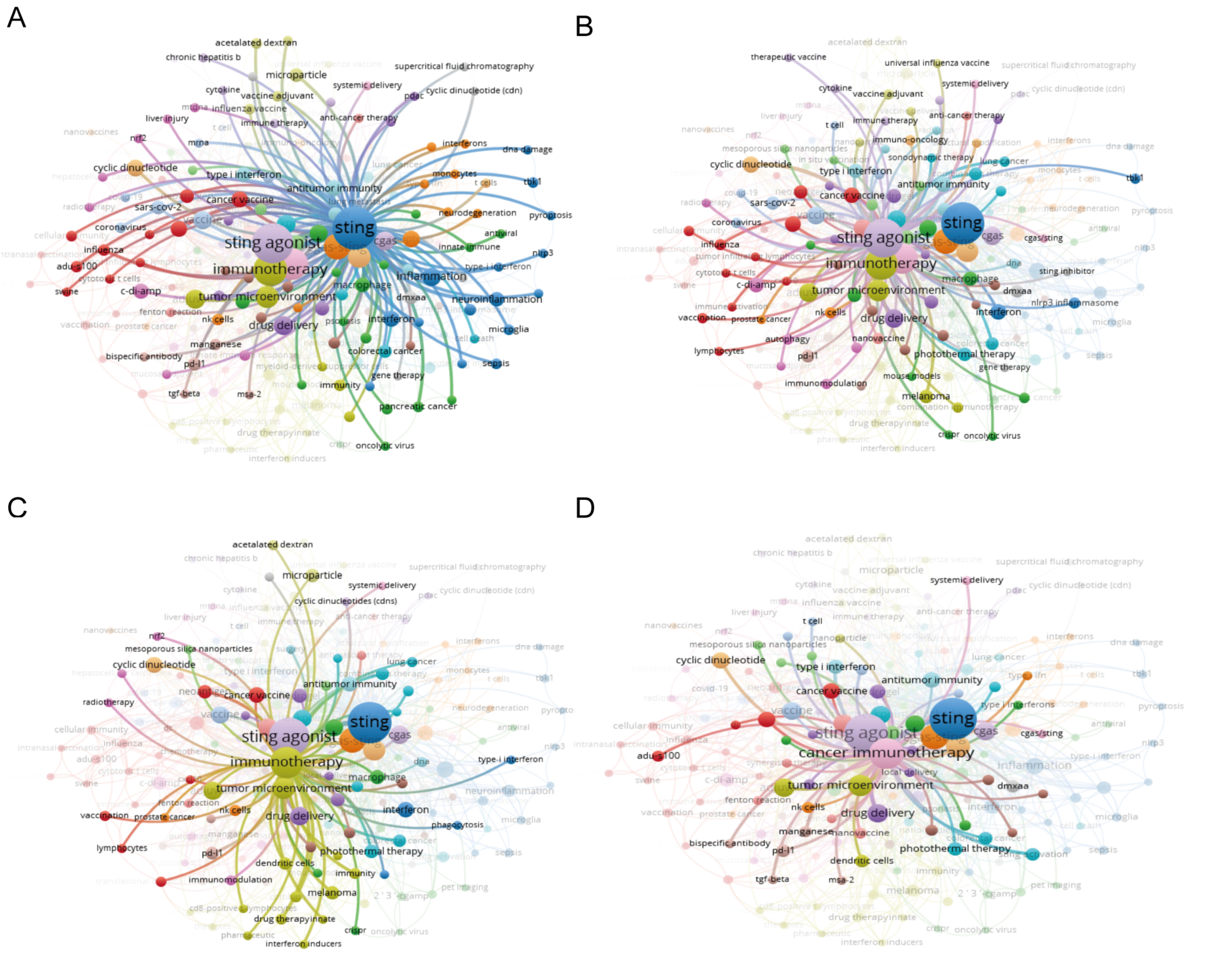

Supplementary Figure 7 (A) Co-occurrence keywords associated with "sting"; (B) Co-occurrence keywords associated with "sting agonist"; (C) Co-occurrence keywords associated with "immunotherapy"; (D) Co-occurrence keywords associated with "cancer immunotherapy".
